# Nomogram to predict the risk of adverse outcomes in patients with residual stones following percutaneous nephrolithotomy

**DOI:** 10.1590/S1677-5538.IBJU.2023.0111

**Published:** 2023-06-25

**Authors:** Feng Xie, Shidong Deng, Kuilin Fei, Hanfeng Xu, Huihui Zhang

**Affiliations:** 1 University of South China The First Affiliated Hospital Hengyang Medical School Hengyang Hunan China Department of Urology, The First Affiliated Hospital, Hengyang Medical School, University of South China, Hengyang, Hunan, China; 2 The First Hospital of Changsha Emergency Department Changsha Hunan China Emergency Department, The First Hospital of Changsha, Changsha, Hunan, China; 3 Central South University Xiangya Hospital Department of Obstetrics Changsha Hunan China Department of Obstetrics, Xiangya Hospital, Central South University, Changsha, Hunan, China

**Keywords:** Nephrolithotomy, Percutaneous, Urinary Calculi, Nomograms

## Abstract

**Purpose:**

To investigate the risk factors associated with adverse outcomes in patients with residual stones after percutaneous nephrolithotomy (PCNL) and to establish a nomogram to predict the probability of adverse outcomes based on these risk factors.

**Methods:**

We conducted a retrospective review of 233 patients who underwent PCNL for upper urinary tract calculi and had postoperative residual stones. The patients were divided into two groups according to whether adverse outcomes occurred, and the risk factors for adverse outcomes were explored by univariate and multivariate analyses. Finally, we created a nomogram for predicting the risk of adverse outcomes in patients with residual stones after PCNL.

**Results:**

In this study, adverse outcomes occurred in 125 (53.6%) patients. Multivariate logistic regression analysis indicated that the independent risk factors for adverse outcomes were the diameter of the postoperative residual stones (P < 0.001), a positive urine culture (P = 0.022), and previous stone surgery (P = 0.004). The above independent risk factors were used as variables to construct the nomogram. The nomogram model was internally validated. The calculated concordance index was 0.772. The Hosmer– Lemeshow goodness-of-fit test was performed (P > 0.05). The area under the ROC curve of this model was 0.772.

**Conclusions:**

Larger diameter of residual stones, positive urine culture, and previous stone surgery were significant predictors associated with adverse outcomes in patients with residual stones after PCNL. Our nomogram could help to assess the risk of adverse outcomes quickly and effectively in patients with residual stones after PCNL

## INTRODUCTION

Percutaneous nephrolithotomy (PCNL) has been recommended as the primarily modality or gold standard for renal stones more than 2 cm, with a high success rate (1, 2). Nevertheless, approximately 15%- 25% of patients with large stones are left with stone fragments after initial PCNL (3). Residual stones less than 4 mm (or sometimes 5 mm), without urinary tract infection or obstruction, are typically referred to as clinically insignificant residual fragments (CIRF) and usually do not require special treatment (4). Tchey et al. also reported that stones can be eliminated naturally through lifestyle changes or medication when residual stones are less than 6 mm in diameter (5).

However, the latest studies have demonstrated that in some patients, small residual fragments or even CIRF are not expelled spontaneously after surgery and can lead to stone-related symptoms, including infection, hematuria, and back pain, and may require surgical treatment (6–9). In addition, the probability of stone growth in patients with upper urinary tract stones after surgery is 10% within 1 year, 35% within 5 years, and up to 50% within 10 years (10, 11). Consequently, the necessity of treating residual stones remains controversial.

Nomogram is a pictorial representation of a complex mathematical formula, employed for calculating probabilities of clinical events in the medical field. In recent years, it has been mentioned in various fields of researches including urolithiasis (12). For example, it can be used to predict urinary leakage complications after PCNL, as well as stone-free status following extracorporeal shock wave lithotripsy in pediatric patients (13, 14). However, to date, no study has focused on developing a nomogram to predict the risk of adverse outcomes in patients with residual stones following PCNL.

Therefore, this study aimed to investigate the risk factors related to adverse outcomes in patients with residual stones after PCNL and to construct a predictive model of adverse outcomes.

## MATERIAL AND METHODS

### Study design

We collected the clinical data of all patients who underwent PCNL for upper urinary tract stones at First Affiliated Hospital of University of South China between January 1st, 2013, and May 31st, 2020. The inclusion criteria were as follows: (1) Patients who had residual stones after PCNL were included, (2) The postoperative residual stones were less than 7 mm in diameter. The exclusion criteria were as follows: (1) patients with incomplete clinical data; (2) patients lost to follow-up; (3) patients with malignant tumors; and (4) patients with obvious ureteral stenosis. A total of 233 cases were ultimately included according to the inclusion and exclusion criteria. The adverse outcome of residual stones was defined as an increase in the diameter of postoperative stones or the occurrence of stone-related symptoms, such as ipsilateral low back pain, fever, and gross hematuria, demanding rehospitalization (15). According to the presence or absence of adverse outcomes of postoperative residual stones, the patients were divided into an adverse outcome group (n =125) and a control group (n = 108). Factors such as sex, age, body mass index (BMI), stone location (left or right), maximum stone diameter, serum creatinine, calcium in blood, urine pH value, maximum diameter of residual stones, urine bacterial and fungal culture, previous stone surgery, hypertension, and diabetes were compared between the two groups. All patients with positive urine cultures were treated with appropriate antibiotics for at least one week and underwent surgery only when the urine culture was negative. The other patients were treated with antibiotics 30 minutes before the beginning of the operation to prevent infection. All patients were treated by performing minimally invasive percutaneous nephrolithotomy (mPCNL) with an access sheath of 18F using a holmium laser for lithotripsy (No. SRM-H3B, Shanghai Ruikeen Laser Technology Co., Ltd.) for upper urinary tract stones. The holmium laser power was set as 50 W. At the end of the procedure, a 16 Fr drainage tube was inserted as a nephrostomy catheter in each patient. The study was approved by the Ethics committee of the First Affiliated Hospital of University of South China (IRB NO. 2022ll0114002). All the experiment protocol involving humans was in accordance to guidelines of institutional and Declaration of Helsinki. Informed consent was received from all patients included in this study.

### Data collection

All patients were diagnosed with renal or upper ureteral calculi by urinary computed tomography (CT) before the operation, and all patients were reexamined by urinary CT examination within 48 hours postoperatively. The maximum diameter of the stone was measured using CT by two radiologists at our institution. The average value of the two measurements was taken if the difference was within 1 mm; if the difference exceeded 1 mm, a third professional remeasured the stone diameter, and the average value of the two closest measurements was used. All patients were followed at least every 6 months during the first year and annually thereafter. Follow-up CT scans were typically performed annually or when a symptomatic episode occurred. Confirmation of an increase in residual stones and the incidence of stone- related symptoms was obtained by interview and CT examination. The rest of the clinical data were from the archived data of the medical records department of our institution.

### Statistical Analysis

Data were analyzed using the software Statistical Package for Social Science version 25.0 (IBM, USA). The continuous variables were calculated as the mean ± SD and analyzed by a two-sample t test. The chi-square test was used to analyze the categorical variables. In multivariate analysis, binary or multiple logistic regression analysis was used, and P values <0.05 were considered statistically significant. According to the results of the multivariate logistic regression analysis model, the nomogram prediction model was created using the R software 4.1.2 (R Foundation for Statistical Computing, Vienna, Austria) rms package. Then, the bootstrap method was used for repeated sampling 1,000 times to internally validate the nomogram model, and a discrimination test was performed by calculating the consistency index (C-index) and area under the curve (AUC), drawing the calibration curve, and performing the Hosmer–Lemeshow goodness-of-fit test to assess the model accuracy.

## RESULTS

Of the 233 patients with residual stones after PCNL for upper urinary tract calculi, adverse outcomes occurred in 125 patients (53.6%), including 64 males (51.2%) and 61 females (48.8%), with a mean age of 50.58 ± 11.39 years. Among them, 18 cases (14.4%) only had an increase in stone diameter without clinical symptoms, and 105 cases (85.6%) had stone-related symptoms. The median time post-PCNL until the development of adverse outcomes was 33.60 months (interquartile range [IQR] 20.50-48.75). There were 108 patients with no adverse outcomes (46.4%), including 70 male patients (64.8%) and 38 female patients (35.2%), with an average age of 53.01 ± 13.24 years. The median clinical follow-up period was 40.47 months (IQR 24.00-59.75).

Table-1 presents the univariate analysis of variables between the two groups of patients. Compared with the control group, more patients with positive urinary cultures (P < 0.001) and longer maximum diameter of residual stones (3.42 ± 1.61 vs. 2.12 ± 1.41, P < 0.001) were found in the adverse outcome group. Female patients had a higher incidence of adverse outcomes than male patients (P < 0.05). Among patients with previous urinary stone surgery, the incidence of adverse outcomes of patients with residual stones after PCNL was higher in the adverse outcome group than in the control group (P = 0.002). Patients with residual stones in the renal pelvis or ureter had a higher rate of the adverse outcome than those with residual stones in the lower calyx (P = 0.026). Notably, there were no significant differences in age, maximum stone diameter, stone laterality, serum creatinine, calcium in blood, urine pH value, BMI, hypertension, or diabetes between the two groups.

**Table 1:** Univariate analysis of variables between the two groups of patients.

Variables		Adverse outcome group (n = 125)	Control group (n = 108)	Statistical value	P
Age (years)		50.58 ± 11.39	53.08 ± 13.24	t = 1.554	0.122
Maximum stone diameter (cm)		2.03 ± 0.91	1.95 ± 0.88	t = -0.668	0.505
Serum creatinine (µmol/L)		97.14 ± 39.47	104.81 ± 53.31	t = 1.258	0.210
Calcium in blood (mol/L)		2.23 ± 1.96	2.21 ± 1.12	t = -0.689	0.386
Urine pH value		6.07 ± 0.70	6.09 ± 0.74	t = 0.031	0.763
Residual stone size (mm)*		3.42 ± 1.61	2.12 ± 1.41	t = 6.475	< 0.001
BMI (kg/m^2^)		23.49 ± 3.49	22.76 ± 3.36	t = -1.622	0.106
**Hypertension**					
	with/without	90/35	79/29	X^2^ = 0.038	0.845
**Diabetes**					
	with/without	110/15	99/9	X^2^ = 0.843	0.359
**Stone laterality**					
	left/right	66/59	49/59	X^2^ = 1.283	0.258
**Gender**					
	male/female	64/61	70/38	X^2^ = 4.395	0.036
**Urine culture**					
	positive/ negative	49/76	16/92	X^2^ = 17.128	< 0.001
**Previous stone surgery**					
	Yes/No	51/74	24/84	X^2^ = 9.161	0.002
Residual stone location, n (%)				X^2^ = 9.242	0.026
Lower pole calix		58(46.4) a	68(63.0) a		
Middle pole calix		40(32.0) a, b	29(26.9) a, b		
Upper pole calix		14(11.2) a, b	8(7.4) a, b		
Renal pelvis/ureter		13(10.4) b	3(2.8) b		

* Residual stone size refers to its maximum diameter.

a, b = No significant difference between the same superscripts.

Subsequently, the variables with p < 0.05 in the univariate analysis were enrolled in the stepwise multivariable logistic regression model based on maximum likelihood estimation. The risk factors such as residual stone size, urine culture, sex, previous stone surgery, and residual stone location were incorporated into this model. Finally, the results revealed that the independent risk factors included residual stone size (OR = 1.707, P < 0.001), positive urine culture (OR = 2.322, P = 0.022), and previous stone surgery (OR = 2.548, P = 0.04) (Table-2).

**Table 2:** Multivariate logistic regression analysis of risk factors for the adverse outcome in patients with residual stones after PCNL.

Variables	Category	OR	95%CI	*P*
Residual stone size (mm)	–	1.707	1.370 – 2.127	<0.001
Urine culture	positive/ negative	2.322	1.132 – 4.765	0.022
Previous stone surgery	Yes/No	2.548	1.341 – 4.841	0.004

Abbreviations: OR = odds ratio; CI = confidence interval

**Figure 1:**
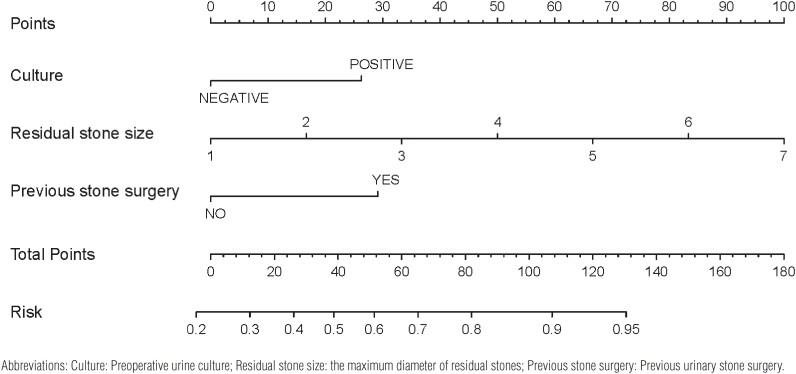
Nomogram for predicting the probability of the adverse outcome in patients with residual stones after PCNL.

**Figure 2:**
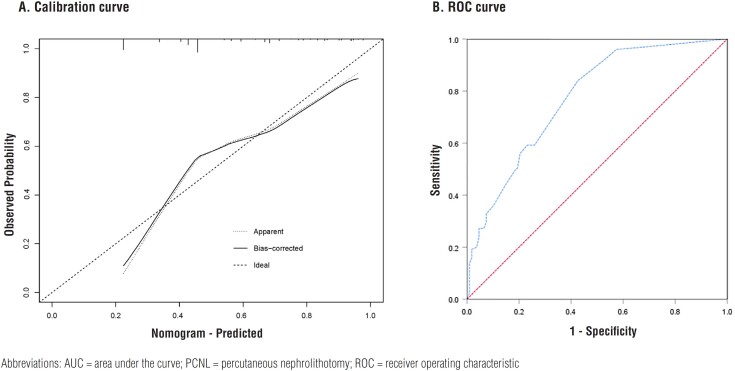
Evaluation of the predictive performance. (A) Calibration curve of prediction model for the adverse outcome in patients with residual stones after PCNL. (B) ROC curve of prediction model for the adverse outcomes in patients with residual stones after PCNL (AUC = 0.772)

Based on the above results, we incorporated significant variables from the multivariate logistic regression analysis into R software to construct a nomogram (Figure-1). The bootstrap method was repeated 1000 times to verify the nomogram model internally. The superiority of the prediction model was tested by the Hosmer-Lemeshow goodness-of-fit test (P = 0.055 > 0.05) and calibration curve (Figure- 2A). The calibration curve of the nomogram model was close to the standard curve. The calculated C- index was 0.772. The effectiveness of the prediction model was further evaluated by the area under the ROC curve (Figure-2B). The area under the ROC curve was 0.772 (95% CI: 0.712 - 0.832), indicating that the model exhibits high accuracy in predicting the adverse outcome of residual stones after PCNL.

## DISCUSSION

Upper urinary tract calculi are one of the most common diseases worldwide. In the past 30 years, the incidence of upper urinary tract calculi has been increasing continuously (16). The harm of upper urinary tract calculi to the human body is significant (17, 18). Although PCNL is one of the primary methods for the treatment of upper urinary tract calculi, PCNL cannot completely eliminate stone fragment residues. The primary aim of our study was to determine the risk factors associated with adverse outcomes in patients with residual stones after PCNL and to establish a nomogram based on these risk factors. This approach represents a completely new logistic regression analysis of patients with residual stones after PCNL, and the risk factors can be obtained easily to assess the risk of adverse outcomes and provide appropriate follow-up plans and early treatment.

Based on previous studies (19, 20), we have concluded that postoperative residual stones may have the following hazards: (1) Postoperative residual stones may cause and perpetuate urinary tract infection. (2) New stones may form with residual stones as the core. (3) The movement of residual stones after surgery may cause obstruction and trigger new symptoms. A study revealed that stones larger than 7 mm exhibited a reduced rate of spontaneous elimination and are more likely to cause clinical symptoms or require surgical intervention (21). If the residual stone size following PCNL exceeded 7 mm, the operation was considered incompletely successful, and further treatment would be necessary. Consequently, this study exclusively included patients with residual stones measuring less than 7 mm in diameter. Although many hazards can be caused by postoperative residual stones, it remains difficult to completely remove all stones due to limitations in surgical equipment, medical technology, and surgical experience. In the past, CIRF was not considered to require specific treatment. However, in recent years, the significance of these residual stones has become controversial. Olvera-Posada et al. evaluated 44 patients with residual stones after PCNL and found that 43% of patients had a stone-related event, with most of them undergoing surgical intervention (3). Osman et al. conducted a similar study, with a median follow-up of 36.2 months. Thirty-three percent of their patients experienced an increase in residual stone size (22). Therefore, in the future treatment of upper urinary tract stones, removing as many residual stone fragments as possible may become a new challenge for urologists. In this study, we found that residual stone size was an independent risk factor for predicting adverse outcomes in patients with residual stones after PCNL. Larger postoperative residual stone sizes were associated with a greater possibility of adverse outcomes, highlighting the necessity of clinical intervention for relatively large postoperative residual stones.

Although there are controversies regarding the treatment of postoperative residual stones, we believe it is important for urologists to help every stone patient eliminate all stones completely in the future. Many researchers have developed novel techniques for the treatment of postoperative residual stones. Friedlander et al. developed a novel device that captures stones in a sealed polyethylene bag in vivo to prevent dispersion of stone fragments during PCNL. The authors reported that the use of this device significantly reduced the median time for stone fragmentation (23). Tan et al. developed a magnetic tool to improve the efficiency of lithotripsy under a ureteroscope. Compared with the traditional Ni-Ti alloy stone extraction basket, the time for magnetic stone extraction tools to extract residual stone fragments is reduced by 53%. Although the visual field of magnetic stone extraction tools is worse than that of traditional stone extraction tools, this design is under further development and improvement (24, 25). With the emergence, innovation, and application of new technologies, the treatment of residual stones after upper urinary tract stone surgery will become much easier in the future of Urology.

In this study, we also found that bacterial or fungal infection in urine was an independent risk factor for adverse outcomes in patients with residual stones after PCNL. Among the cultured bacterial species, Escherichia coli was the most common, with 27 (55.1%) cases in the adverse outcome group and 10 (58.8%) cases in the control group. Previous studies have shown that in Escherichia coli infected kidneys, the expression of osteopontin is elevated in renal tubular epithelial cells, which leads to tubular epithelial cell damage and induces a renal inflammatory response that activates multiple immune response signaling pathways, thereby promoting stone formation (26). Furthermore, a study reported that urinary tract infections and stones potentiate each other (27). Therefore, to reduce the risk of adverse outcomes in patients with residual stones after PCNL, urinary tract infections should be avoided whenever possible.

Previous stone surgery was another risk factor. Similarly, Keskin analyzed 417 patients undergoing urinary surgery and found that readmission rates were significantly higher in patients with previous stone treatments than in patients with no history of stone treatments (9). Patients with a history of previous stone surgery are also at an increased risk of stone recurrence after another subsequent stone procedure (except for second stage surgery), likely because they are already recurrent stone formers. These patients may possess certain undetected risk factors, such as metabolic status, genetic factors, and eating habits, leading to the recurrence of stones, which eventually will require intervention (28). The specific mechanism involved deserves further study.

Based on the above three independent risk factors, residual stone size, positive urine culture, and previous stone surgery, we constructed a nomogram to predict the probability of adverse outcomes. Through internal verification, the C- index of the nomogram was 0.778, indicating a good consistency. The risk factors can be easily obtained. This prediction model can not only help in clinical decision-making but also provides a visual tool to assess the incidence of adverse outcomes in patients with residual stones after PNCL.

However, several limitations of this study must be highlighted: (1) The sample size of cases was relatively small. (2) The results were derived from a retrospective analysis, and a certain degree of selection bias was present. (3) This was a single-center study, and a multicenter study should be performed to further verify our results.

## CONCLUSION

In conclusion, a larger residual stone diameter, positive urine culture, and previous stone surgery were significant predictors associated with adverse outcomes in patients with residual stones after PCNL. Our nomogram could help to simply and effectively assess the risk of adverse outcomes in patients with residual stones after PCNL.
